# Honey bee (*Apis mellifera*) larval pheromones may regulate gene expression related to foraging task specialization

**DOI:** 10.1186/s12864-019-5923-7

**Published:** 2019-07-19

**Authors:** Rong Ma, Juliana Rangel, Christina M. Grozinger

**Affiliations:** 1 Department of Entomology, Center for Pollinator Research, Center for Chemical Ecology, Huck Institutes of the Life Sciences, Pennsylvania State University, University Park, PA USA; 2 Department of Entomology, Texas A&M University, College Station, TX USA

**Keywords:** Animal behavior, Behavioral plasticity, Communication, Differential gene expression, Gene networks, Larval pheromone signals, Task specialization

## Abstract

**Background:**

Foraging behavior in honey bees (*Apis mellifera*) is a complex phenotype that is regulated by physiological state and social signals. How these factors are integrated at the molecular level to modulate foraging behavior has not been well characterized. The transition of worker bees from nursing to foraging behaviors is mediated by large-scale changes in brain gene expression, which are influenced by pheromones produced by the queen and larvae. Larval pheromones can also stimulate foragers to leave the colony to collect pollen. However, the mechanisms underpinning this rapid behavioral plasticity in foragers that specialize in collecting pollen over nectar, and how larval pheromones impact these different behavioral states, remains to be determined. Here, we investigated the patterns of gene expression related to rapid behavioral plasticity and task allocation among honey bee foragers exposed to two larval pheromones, brood pheromone (BP) and (E)-beta-ocimene (EBO). We hypothesized that both pheromones would alter expression of genes in the brain related to foraging and would differentially impact brain gene expression depending on foraging specialization.

**Results:**

Combining data reduction, clustering, and network analysis methods, we found that foraging preference (nectar vs. pollen) and pheromone exposure are each associated with specific brain gene expression profiles. Furthermore, pheromone exposure has a strong transcriptional effect on genes that are preferentially expressed in nectar foragers. Representation factor analysis between our study and previous landmark honey bee transcriptome studies revealed significant overlaps for both pheromone communication and foraging task specialization.

**Conclusions:**

Our results suggest that, as social signals, pheromones alter expression patterns of foraging-related genes in the bee’s brain to increase pollen foraging at both long and short time scales. These results provide new insights into how social signals and task specialization are potentially integrated at the molecular level, and highlights the possible role that brain gene expression may play in honey bee behavioral plasticity across time scales.

**Electronic supplementary material:**

The online version of this article (10.1186/s12864-019-5923-7) contains supplementary material, which is available to authorized users.

## Background

One of the hallmarks of insect sociality is division of labor, whereby group members specialize on different tasks that are essential to group survival and reproduction [1, 2]. Understanding the proximate and ultimate mechanisms mediating social behavior, division of labor, and task specialization is a major focus of behavioral sociobiology [3–8]. Several studies have clearly demonstrated that complex animal behaviors, including social interactions, are regulated by transcriptional, neural, and physiological networks [9–12]. Moreover, several studies have suggested that behavioral ontogeny is mediated by differential regulation of core, well-conserved transcriptional or physiological “toolkits” that regulate behavioral modules [4, 13–19]. However, the mechanisms mediating more rapid shifts in behavior and task specialization have not been examined as thoroughly [20–22].

As in many social insects, honey bee (*Apis mellifera*) workers exhibit a form of age-based task allocation in which behavioral repertoires incrementally expand or shift over the course of an individual’s lifetime [23]. This phenomenon—called age-based polyethism—is regulated both genetically and environmentally, and provides a tractable system in which to investigate temporal dimensions of behavioral plasticity [24, 25]. Honey bees spend the first weeks of their lives performing tasks within the relative safety of the hive, including tending to the needs of developing larvae (i.e., nursing), before transitioning to increasingly dangerous tasks near the nest entrance and beyond, including foraging [26]. Once they begin to forage, workers may further specialize by collecting predominantly one floral resource type (either pollen or nectar [27]), and their proclivity for pollen vs. nectar foraging can persist throughout their lives. Bees that specialize on nectar vs. pollen foraging exhibit distinct behavioral, physiological, and transcriptional traits. For example, upon returning to the colony, nectar foragers regurgitate collected nectar to nestmates waiting to process it, while pollen foragers pack their pollen loads into honeycomb themselves [28, 29]. Nectar and pollen foragers also differ in their neural and sensory responses to sugar [30] and pheromones [31, 32].

Pheromone communication in honey bees plays a key role in mediating behavioral transitions across time scales [9, 33–36]. Pheromones are typically categorized by the time scale at which they induce behavioral changes in receivers: primer pheromones cause slow, enduring changes in physiology, while releaser pheromones cause rapid, ephemeral responses. Primer pheromones generate long-term changes in behavior and physiology by altering patterns in gene expression, especially in the brain [9, 33–36]. For example, brood and queen pheromones delay the behavioral transition from nurses to foragers by altering the expression of large numbers of genes in worker brains [33, 36]. In contrast, releaser pheromones elicit rapid behavioral changes either by activating or modulating neural circuits, triggering molecular signaling pathways, or regulating gene expression [34, 37–39]. For example, the alarm pheromone in honey bees elicits aggressive behaviors against intruders by activating the expression of immediate early genes in the brain [34], while one component of queen pheromone, homovanillyl alcohol, elicits grooming behavior from workers by binding to an olfactory receptor in the antennae, activating dopamine receptors in the brain, and regulating brain gene expression [33, 40, 41].

Honey bee larval pheromones cause primer and releaser effects that blur the distinction between these categories, which provides a fascinating opportunity to understand regulation of behavior across time scales. Two larvae-produced pheromones, brood pheromone (BP) and (E)-beta-ocimene (EBO), have been shown to elicit rapid increases in pollen foraging within an hour of exposure and lasting for 3 hours [42]. Both pheromones are produced by developing larvae but differ in the timing of their peak production, such that EBO is produced early in larval development while BP is produced later on, just before pupation [42]. Both larval pheromones cause additional behavioral and physiological effects in honey bee workers. In fact, brood pheromone induces the greatest number of known primer responses in honey bees, including modulation of sucrose response thresholds, ovary development, foraging ontogeny, foraging choice behavior, and hypopharyngeal gland development [43]. The effect of brood pheromones on forager behavior seems to be driven by an increase in pollen foraging. Specifically, brood pheromones cause an increase in the number of foraging trips and the size of pollen loads [42, 44], and this effect is not driven by task-switching from nectar to pollen foraging [42]. Both pheromones also increase the size of the foraging force of the colony in the long term, accelerating the transition of bees from performing within-hive roles to foraging [44–46]. Interestingly, some components of EBO and BP are also produced by honey bee adults as well. For example, EBO is also produced by mated queens [47], and foragers produce ethyl oleate, a component of BP [48]; both impact the ontogeny of foraging behavior [48, 49]. Queens and larvae both produce another BP component, ethyl palmitate, which inhibits ovarian development [37]. Although BP components are also produced in adults, the full blend of BP and EBO has only been described in honey bee larvae, and multi-component pheromone blends often have synergistic effects [37]. Overall, larval pheromones have a strong effect on pollen foraging but not nectar foraging in the short term (i.e., hours), and they are also involved in regulating the size of the foraging labor force in the long term (i.e., weeks).

Chronic exposure of honey bee adults to pheromones that cause primer effects, including BP, have been shown to affect the expression of genes involved in methylation and chromatin remodeling [50]. However, it is unclear if similar epigenetic effects are observed when pheromones act at the short-term, releaser time scale. This is a fascinating system because both pheromones (BP and EBO) regulate foraging behavior, but at different temporal scales. How these behavioral transitions across different temporal scales are related, or how their underlying genetic, epigenetic, and physiological mechanisms interact to regulate foraging behavior, remains to be determined.

In previous studies, the effects of BP on gene expression were evaluated on whole brain expression patterns from bees collected at five and fifteen days of age, after life-long exposure to brood pheromone [36]. However, in that study, the bees were collected without regard to their behavior, including their foraging preference. Consequently, we seek to more precisely characterize the transcriptional differences associated with rapid, pheromonally-regulated changes in honey bee foraging, and to juxtapose these rapid changes with more stable differences in gene expression associated with task specialization, specifically in integration centers of the brain (i.e., mushroom bodies). Given that foragers have similar behavioral responses to BP and EBO [51], we hypothesized that these two pheromones regulate a common set of foraging genes in the brain (i.e., a foraging “toolkit”). Because BP and EBO have more pronounced effects on pollen foraging than nectar foraging [42, 45], we further hypothesized that larval pheromones affect foragers differentially depending on foraging task specialization. We thus compared the effects of EBO and BP exposure on foragers previously found to specialize on nectar or pollen to test the following four predictions: 1) foragers specializing on pollen vs. nectar foraging exhibit distinct patterns of gene expression in the brain, 2) BP and EBO stimulate the same transcriptional profiles in the brains of forager bees, 3) changes in the same behavior at different time scales (i.e., transition to and/or stimulation of pollen foraging) utilize similar molecular mechanisms, and 4) both larval pheromones have more pronounced effects on gene expression in pollen foragers than nectar foragers.

Combining differential gene expression, clustering, and network analyses, our study presents several lines of evidence that support the predictions of the hypothesis that larval pheromones regulate a common suite of genes involved in foraging tasks. Specifically, nectar and pollen foragers showed distinct patterns of brain gene expression, BP and EBO do regulate a common set of genes, and changes in short-term and long-term shifts in foraging behavior are regulated by similar sets of genes. The results of the study did not support the hypothesis that larval pheromones affect gene expression more strongly in pollen foragers than nectar foragers, however. Contrary to our prediction, the data showed that exposure to larval pheromones produced gene expression profiles that significantly overlapped with those of nectar foragers but not pollen foragers. Our study provides insights into the molecular mechanisms underlying task allocation in honey bees, and highlights the possible role that brain gene expression plays in behavioral plasticity across time scales. It also probes the interface between ephemeral and more consistent changes in behavior to gain insight into mechanisms that permit behavioral plasticity and complexity across time.

## Results

### Transcript quantification

**Fig. 1:**
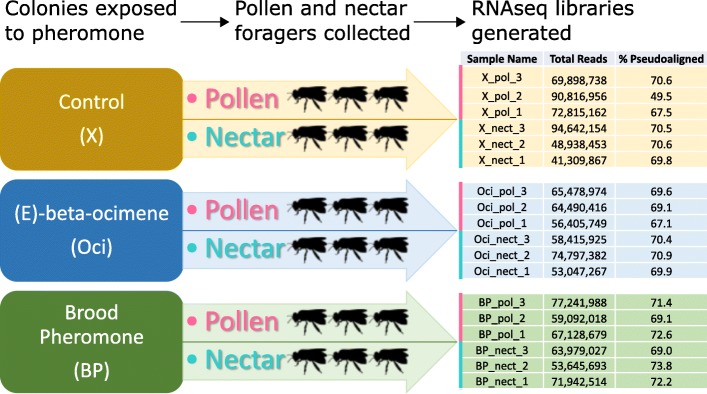
Overview of experimental design and sequencing. RNA-seq libraries were generated from nectar and pollen foragers exposed to three pheromone treatments. Three pooled pollen forager samples and three pooled nectar forager samples were collected for each pheromone treatment. Each bee diagram represents a sample, though two brains were used for each sample. Resulting numbers of reads per sample and percentages of those reads that mapped to the honey bee genome are presented in a table to the right

The RNA samples collected in this study were extracted from mushroom bodies of pollen and nectar foragers exposed to one of three pheromone treatments: paraffin oil control, brood pheromone (BP), or E-beta-ocimene (EBO) (Fig. 1). The number of RNA-seq reads per sample ranged from 41 to 94 million, with an average of 65 million reads per sample. After quality filtering and adapter trimming, an average of 69% of the reads per sample were pseudoaligned to generate transcript abundance for each annotated transcript in the recently updated honey bee genome annotation (Amel_HAv3.1; Additional file 1: Table S1). Overall, 9179 genes were detected in all samples and were included in subsequent analyses, representing 74% of the 12,332 annotated honey bee genes.

### Differential gene expression

Differential gene expression analysis was performed to characterize the effects of pheromone treatment, forager-type, and the interaction between pheromone and forager type. There were 533 differentially expressed genes (DEGs) whose expression varied in at least one contrast (FDR < 0.05), including 269 DEGs related to pheromone treatment and 326 DEGs related to forager type (Table 1; Additional file 2: Table S2). Additionally, there were 131 DEGs that showed a statistically significant interaction between forager type and pheromone treatment. The lists of all DEGs are provided in Additional file 2: Table S2.

**Table 1:** Numbers of DEG in all pairwise comparisons

		Upregulated	Downregulated
Pheromone Main Effect	BP vs Control	12	46
	EBO vs Control	14	138
Food Main Effect	Pollen vs Nectar	79	246
Interaction Effect	BP v Control and Food	29	39
	EBO v Control and Food	55	32

Genes whose expression differed between groups were considered differentially expressed when they had a false discovery rate (FDR) of <0.05. Up- and down- regulation of significantly differentially expressed genes was determined by whether log fold change was above or below zero, respectively

Of the 269 DEGs related to pheromone treatment (pheromone-related DEGs), there were 58 DEGs between BP and control samples, and 152 DEGs between EBO and control samples, indicating that EBO’s effect on gene expression was almost three times greater than that of BP. In addition, there were 148 genes that showed differences between BP and EBO samples. Because there were many genes that were differentially expressed in more than one contrast, we performed hypergeometric tests to further determine if there were more shared DEGs than those from random expectation among pheromone treatments, and between pheromone treatments and forager type. There were significant overlaps between all pairwise comparisons of pheromone treatment, indicating that BP and EBO regulate expression of a common subset of genes or genetic pathways (Table 2).

**Table 2:** Overlaps between pheromone-related DEG

First Contrast	Second Contrast	DEGs in First Contrast	DEGs in Second Contrast	Overlap
BP vs Control	EBO vs Control	58	152	39*

There was a significant overlap between BP-related DEGs and EBO-related DEGs in a hypergeometric test

*significantly greater overlap of genes than expected by chance; *P* < 0.001; hypergeometric test

Pheromone-related DEGs were then compared to DEGs that differed between nectar and pollen foragers (foraging-related DEGs). While we found significant overlaps between foraging-related and pheromone-related DEGs (Table 3), it is important to note that nectar vs. pollen foraging was a binary trait, so genes that were upregulated in one foraging context were necessarily downregulated in the opposite foraging context. For example, genes that were upregulated in pollen foragers were also downregulated in nectar foragers, and vice-versa. To further explore these results, we split the foraging-related DEGs into those that were upregulated in pollen foragers (and thus downregulated in nectar foragers) and those that were upregulated in nectar foragers (and thus downregulated in pollen foragers), and again looked for overlaps with DEGs from each pheromone treatment. Interestingly, there were significant overlaps between pheromone-related DEGs and DEGs upregulated in nectar foragers (Table 4; hypergeometric tests, *p* < 0.01), but not between pheromone-related DEGs and DEGs upregulated in pollen foragers. In summary, BP and EBO both regulated foraging-related genes, and this effect was driven primarily by genes upregulated in nectar foragers relative to pollen foragers.

**Table 3:** Overlaps between pheromone- and foraging-related DEG

	Pheromone genes	Foraging Genes	Overlap
BP vs Control	58	386	41*
EBO vs Control	152	386	71*

There was a significant overlap between pheromone-related DEGs and DEGs related to foraging

*significantly greater overlap of genes than expected by chance; *P* < 0.001; hypergeometric test

**Table 4:** Overlaps between pheromone- and foraging-related genes, separated by foraging preference

	Pheromone genes	Pollen Upregulated	Nectar Upregulated	Overlap Pollen	Overlap Nectar
BP vs Control	58	79	246	1	40*
EBO vs Control	152	79	246	0	71*

Because foraging preference was a binary trait in the generalized linear model (i.e. either pollen or nectar), DEGs that were up-regulated in nectar foragers were by definition down-regulated in pollen foragers, and vice versa. Foraging-related DEGs were upregulated in pollen foragers (and downregulated in nectar foragers) when log fold change was greater than 0 and upregulated in nectar foragers (and downregulated in pollen foragers) when log fold change was less than zero. There was a significant overlap between pheromone-related genes and genes that were upregulated in nectar foragers

*significantly greater overlap of genes than expected by chance; *P*<0.001; hypergeometric test

To better understand the function of differentially expressed genes associated with forager type and pheromone treatment, we performed gene ontology (GO) enrichment analysis for DEGs associated with pheromone treatment, forager type, and their interaction. DEGs associated with forager type were significantly enriched for GO terms related to lipid metabolism and trypsin-like serine proteases (FDR < 0.05). DEGs related to pheromone treatment were enriched for integral components of membrane, fatty acid metabolism, and lipid biosynthesis (FDR < 0.05). Finally, DEGs related to the interaction of pheromone treatment and forager type were enriched for lipid biosynthesis and metabolism (FDR < 0.05).

The DEGs associated with either EBO or BP were also analyzed separately. Because there were few upregulated genes associated with either pheromone, up- and down-regulated genes for each pheromone were pooled during pathway enrichment analysis, with the understanding that the results for pheromone could potentially be driven by down-regulated genes. DEGs associated with BP exposure were enriched for lipid biosynthesis and integral components of the membrane (FDR < 0.05). DEGs associated to EBO exposure were enriched for integral components of membrane, fatty acid biosynthetic processes, fatty acid metabolism, and the pentose phosphate pathway. There was a significant overlap of 39 genes between BP and EBO exposed foragers compared to controls (*P* < 0.05), and these DEGs were significantly enriched for metabolic pathways and fatty acid metabolism (FDR < 0.05).

### Hierarchical clustering and principal components analysis (PCA)

**Fig. 2:**
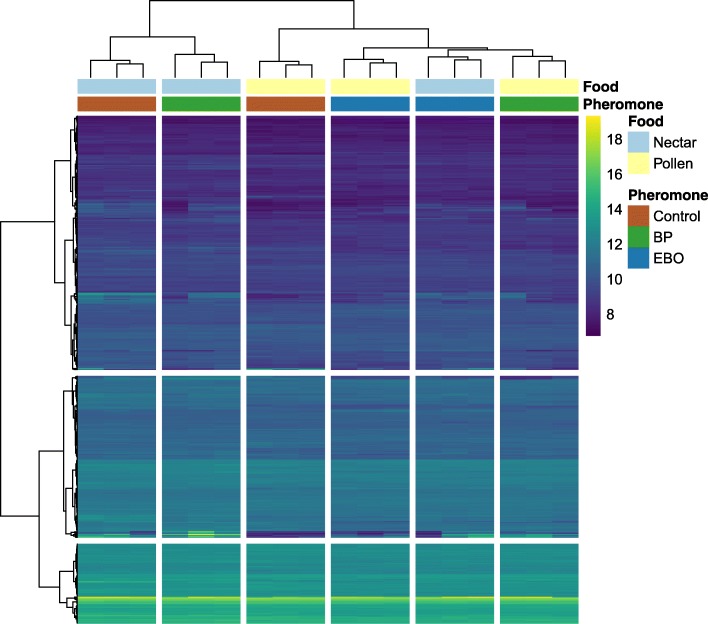
Heatmap for the hierarchical clustering of brain gene profiles. Honey bees foraging on pollen or nectar were exposed to pheromone treatments: Brood pheromone (BP), E-beta-ocimene (EBO), or a control. Rows correspond to differentially expressed genes, and columns represent samples. Food and pheromone treatments for each sample are represented between sample dendrogram and heatmap. The scale bar indicates variance stabilized gene expression values, with highly expressed genes in lighter colors and lower expression in darker colors. Clustering of samples shows two branches main branches, which correspond broadly to nectar foraging (left) and pollen foraging (right); however, nectar foragers exposed to EBO have expression profiles more similar to pollen foragers. Within pollen and nectar branches, there is also a split in pheromone treatments

Hierarchical clustering analysis and PCA were used to better understand broad patterns across all DEGs. Based on all variance-stabilized gene expression values of DEGs, hierarchical clustering grouped samples with identical combinations of pheromone treatment and forager type (Fig. 2) significantly more often than random expectation based on 10,000 iterations of multiscale bootstrap resampling (*P* < 0.05; Additional file 4: Figure S1). Nectar foragers exposed to either BP or control pheromone treatments clustered together. However, nectar foragers exposed to EBO clustered with pollen foragers, suggesting that EBO exposure resulted in gene expression patterns of nectar foragers that were more similar to those of pollen foragers. This is consistent with the observation that EBO had a greater effect on overall gene expression than BP. Pollen foragers exposed to BP or EBO were more similar to each other than either group was to pollen foragers exposed to control treatments. Genes were also clustered based on the similarity of their expression, and several large clusters of genes emerged.

**Fig. 3:**
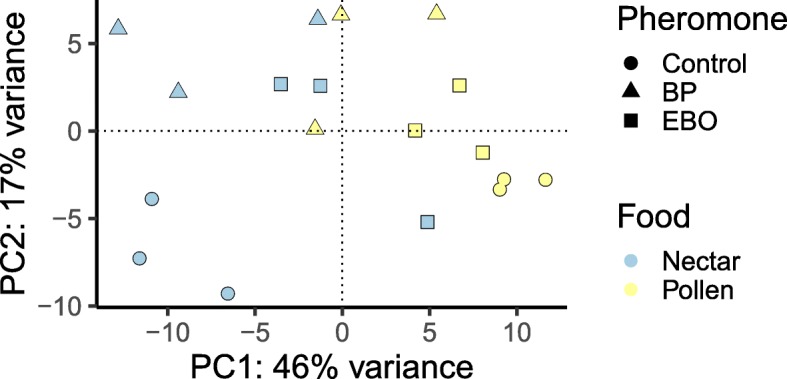
Principal component analysis of all DEG. The first two principal components (PCs) are displayed, together representing 63% of the total variation. Each point represents a single sample. PC1 separates samples based on food preference, whereas PC2 separates pheromone treatment, particularly for nectar foragers. Shape represents pheromone treatment. Color represents pollen or nectar forager-type. The percentage of variation in transcript expression patterns explained by each PC is shown in the axes

To better understand the contributions of pheromone treatment and forager type on patterns of gene expression, we performed PCA on all DEGs with samples grouped by treatment. Each principal component (PC) was composed of a linear combination of many genes. Together, the first two PCs explained 63% of variance in the data, and the PCs were useful in separating samples by both pheromone treatment and forager type (Fig. 3). The first PC explained 46% of variance and separated nectar and pollen foragers, indicating that the greatest axis of variation in gene regulation was related to forager type. This is consistent with results from the differential gene expression analysis, which showed that there were more DEGs associated with forager type than with pheromone exposure. The second PC explained 17% of the variance in the DEGs and began to separate pheromone treatment from each other, although the separation was less distinct than for forager type. Specifically, PC2 seemed to separate bees exposed to control pheromone treatment from those exposed to BP, while samples from bees exposed to EBO were more intermediate. Pollen foragers, especially those exposed to EBO and control treatments, seemed to have a lower variance than nectar foragers in both principal components. PC3 and PC4 explained 14% and 5% of the variance in DEGs, respectively (Additional file 6: Figure S3).

### Overlaps with landmark studies

To explore the relationship between the results shown above and those of previous similar studies, we performed representation factor analysis between our results and landmark honey bee transcriptome studies (Tables 5, 6) [36, 52]. Whitfield et al. [52] identified DEGs related to foraging ontogeny, while Alaux et al. [36]. identified DEGs related to long-term exposure to BP (i.e., primer pheromone effects). We found a significant overlap between the foraging-related DEGs identified in our study and those identified by [52] (hypergeometric test, P < 0.05; Table 6). Thus, genes that were differentially expressed in the brains of nectar and pollen foragers (our study) overlapped significantly with genes that were differentially expressed in nurses and foragers [52]. Similarly, we found a significant degree of overlap (hypergeometric test, P < 0.05) between DEGs associated with BP exposure in our study and BP-related DEGs identified in [36] after 15 days of continuous exposure. Thus, long-term changes in gene expression associated with impacts of BP exposure on the transition from nursing to foraging tasks overlap significantly with short-term changes in brain expression patterns associated with the stimulation of foraging behavior by BP. This ultimately suggests that behavioral plasticity utilizes common suites of genes at vastly different time scales.

**Table 5:** Overlaps between pheromone-related genes and those of Alaux et al

	Genes represented in both	BPgenes	Alaux et al., BP after 5 days	Alaux et al., BP after 15 days	Overlap BP5	Overlap BP15
BP vs Control	6039	49	104	85	1	2*

Alaux et al. [36] used a microarray to characterize brain gene expression differences related to long-term exposure to BP (i.e. primer pheromone effects) at two time points, 5 days (BP5) and 15 days (BP15). Shown here are the results of a hypergeometric test between the DEGs related to BP in the present study and in Alaux et al. at each time point. There was a significant overlap between DEGs in the present study and the 15-day treatment in Alaux et al

*significantly greater overlap of genes than expected by chance; *P* < 0.05; hypergeometric test

**Table 6:** Overlaps between foraging-related genes and Whitfield et al

	Genes represented in both	Foraging-related	Whitfield et al	Overlapping genes
BP vs Control	6039	264	839	48*

Whitfield et al. [52] identified DEGs related to foraging ontogeny during the transition between nurses and foragers, controlling for the effect of age. Presented here are the results of a hypergeometric test between the foraging-related DEGs in the present study and the DEGs identified in Whitfield et al., which show a significant overlap

*significantly greater overlap of genes than expected by chance; *P* < 0. 05; hypergeometric test

### Weighted gene co-expression network analysis (WGCNA)

**Fig. 4:**
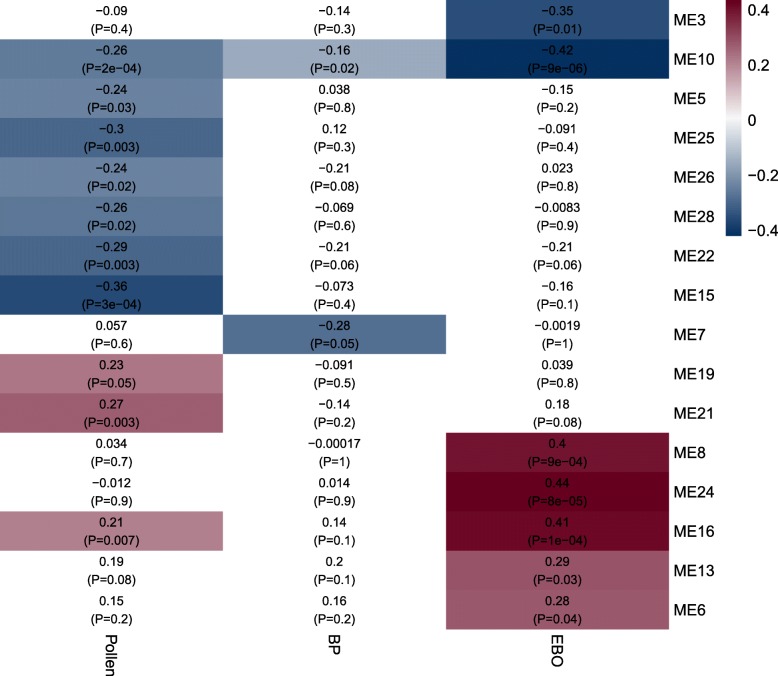
Weighted gene co-expression network analysis. Rows represent gene modules. Columns represent sample traits. Each cell contains two values: a correlation coefficient between the module and sample trait and the associated p-value in parentheses. Significant correlations are colorized according correlation coefficient, varying from high values in yellow to low values in purple

We used WGCNA to construct networks of genes based solely on the similarity of their expression patterns to organize co-expressed genes into groups, called modules. These modules were constructed independently of trait information and were then correlated to traits using a generalized linear model. Specifically, we looked at relationships between each module and three traits of interest: pollen vs. nectar foraging, BP vs. control, and EBO vs. control. The WGCNA identified 16 modules that were significantly correlated to forager type, exposure to BP, exposure to EBO, or a combination thereof (GLM, P < 0.05; Fig. 4). Fourteen modules were significantly correlated with only one trait. Module 10 was the only module that was associated with all traits, while Module 16 was associated with forager type and EBO exposure, but not BP exposure. For each module, the most highly connected gene in the network was identified (Table 7), providing a list of candidate genes. The top five most connected genes for each module can be found in the Additional file 7: Table S4.

**Table 7:** WGCNA Module Hub genes

Regulation Pattern	Module	Size	Hub Gene	Hub Gene Description
EBO & Forager-type	16	166	GB52658	Transcription factor
All: Forager-type, BP, & EBO	10	239	GB45943^a^	Collagen alpha-5 chain
BP Only	7	560	GB42728	Sodium channel protein paralytic
EBO Only	13	217	GB45423	transmembrane protein
	3	900	GB52595	zinc finger and BTB domain-containing protein 20
	8	90	GB45063^a^	LIM/homeobox protein Lhx9
	24	267	GB19920	phosphopantothenoylcysteine decarboxylase
	6	540	GB44289	ataxin-3
Forager-type Only	19	127	GB50923	serine-protein kinase ATM
	21	145	GB49517	DENN domain-containing protein 4C
	22	121	GB51059	four and a half LIM domains protein 2
	15	168	GB45147 ^a^	clavesin-2
	26	82	GB41641^a^	mitochondrial cardiolipin hydrolase
	28	58	GB50931	box A-binding factor
	5	594	GB40539	40S ribosomal protein S20
	25	89	GB51029	band 4.1-like protein 5

^a^hub genes that were also differentially expressed in at least one contrast

To better understand the functions of the gene modules identified in this analysis, we performed Kyoto Encyclopedia of Genes and Genomes (KEGG) pathway analysis on three modules (Table 8). Module 10 was chosen based on its significant correlation with food and both brood pheromones, and modules 3 and 7 were selected based on their strong correlations with BP and EBO, respectively. Module 10 was enriched for KEGG pathways related to metabolic pathways, carbon metabolism, fatty acid metabolism, and peroxisomes (Wilcoxon, *P* < 0.05). Module 7 was significantly enriched for glycerophospholipid metabolism, neuroactive ligand-receptor interaction, and hippo signaling pathway (Wilcoxon, *P* < 0.05). Module 3 was enriched for metabolic pathways like FoxO and AGE-RAGE signaling pathways, development pathways like wnt signaling, and immune pathways like Toll and lmd signaling pathways (Wilcoxon, *P* < 0.05).

**Table 8:** KEGG analysis of selected WGCNA modules

Module	Trait association	Significantly enriched KEGG pathways (*P* < 0.05)	Significantly enriched GO categories (EASE <0.05)
10	BP, EBO, Forager-type	Metabolic pathways	Integral components of membrane, Fatty acid biosynthetic process
		Carbon metabolism	
		Fatty acid metabolism	
		Peroxisome	
7	BP alone	Glycerophospholipid metabolism	Ion channel
		Neuroactive ligand-receptor interaction	
		Hippo signaling pathway	
3	EBO alone	Pentose and glucuronate interconversions	Integral components of membrane
		Metabolic pathways	
		FOXO signaling pathway	
		Neuroactive ligand-receptor interaction	
		Lysosome	
		Wnt signaling pathway	
		Dorso-ventral axis formation	
		Notch signaling pathway	
		Toll and Imd signaling pathway	
		AGE-RAGE signaling pathway in diabetic complications	

Module 10 was chosen based on its significant correlation with food and both brood pheromones, and modules 3 and 7 were selected based on their strong correlations with BP and EBO, respectively

## Discussion

In the present study, we investigated the genes and transcriptional pathways underlying rapid behavioral responses to pheromone signals in honey bee foragers specialized in pollen or nectar foraging. We hypothesized that two larval pheromones, brood pheromone (BP) and E-beta-ocimene (EBO), would regulate a common set of foraging genes in the brain, and that these pheromones would affect gene expression differentially depending on task specialization. We found that nectar and pollen foragers have distinguishable gene expression profiles, and that both larval pheromones do indeed regulate a shared set of genes and transcriptional pathways, supporting predictions 1 and 2, respectively. Moreover, comparisons with previous studies suggest that similar genes regulate the ontogeny of foraging behavior and foraging task specialization, and a common set of genes mediate both short- and long-term responses to BP, supporting prediction 3. However, larval pheromones affected transcriptional pathways more strongly in nectar foragers than pollen foragers, contrary to prediction 4. Therefore, we found support for the hypothesis that larval pheromones regulate a shared set of foraging genes in the brain, and that their effect depends on foraging preference or specialization. However, our results did not support our prediction that larval pheromone have a greater effect on pollen foragers. Instead, the data revealed that larval pheromones regulated genes are positively correlated with nectar foraging. Thus, our study begins to elucidate mechanistic links between larval pheromone communication and foraging specialization and suggests that common transcriptional pathways may regulate behavior across time scales.

The present study demonstrates for the first time that there are transcriptional differences between nectar and pollen foragers in the mushroom bodies of honey bees (prediction 1). Several quantitative trait loci have been identified which underlie colony-level variation in the propensity to collect pollen vs. nectar, and these loci are associated with variation in the sugar concentration of nectar collected and the amount of pollen and nectar brought back to the hive [53, 54]. Previous studies have examined the genetic and behavioral differences associated with preference for nectar vs. pollen foraging [27, 53, 55–57]. In our study, foraging specialization on nectar vs. pollen foraging was associated with substantial differences in gene expression profiles (with almost 400 DEGs; Table 1), and with variation among nectar and pollen foragers, which accounted for 46% percent of the overall variation in DEGs (Fig. 3). To elucidate transcriptional pathways that respond to larval pheromones, we utilized weighted gene correlation network analysis (WGCNA) to provide a more detailed view of the molecular processes associated with traits of interest [58, 59]. WGCNA identified 16 genetic modules that were significantly correlated with foraging or pheromone exposure (Fig. 4), most of which were associated with foraging specialization (Fig. 4).

Short exposure to both BP and EBO significantly altered gene expression profiles in the brains of foragers, and both pheromones regulated overlapping sets of genes (prediction 2). Exposure to EBO was associated with 169 DEGs, which was nearly three times greater than the number of DEGs regulated by BP (Table 1). Yet, even in this limited gene set, there was a statistically significant overlap in the DEGs regulated by BP and EBO (Table 2), and the overlapping genes were enriched for fatty acid metabolism. Hierarchical clustering and principal component analyses confirmed that pheromone exposure had strong and consistent effects on gene expression profiles. Furthermore, WGCNA revealed that module 10, representing 239 genes with correlated expression patterns, was significantly downregulated in samples exposed to either pheromone. Together, these results suggest that BP and EBO regulate overlapping genetic modules and pathways that are enriched for energy metabolism. Decreasing whole-brain energy metabolism, including that of fatty-acids, is associated with long-term behavioral transition from in-hive tasks to foraging tasks [60], suggesting that larval pheromones regulate foraging behavior by specifically activating pathways involved in the natural ontogeny of foraging behavior.

Changes in the same behavior at different time scales, such as the ontogeny of pollen foraging and the pheromonal upregulation of pollen foraging, may utilize similar molecular mechanisms (prediction 3). We reached this intriguing conclusion after comparing our results to those of two landmark honey bee transcriptome studies [36, 52]. Whitfield et al. [52] compared nurses and foragers, controlling for age, and found over 1000 DEGs. Alaux et al. [36] were the first to study the effects of brood pheromone on gene expression, and found more than 200 DEGs between age-matched bees that were exposed to BP continuously for multiple days (i.e., five or 15 days) and those that were not exposed. To test the degree of overlap between our results and those from previous studies, we compared 1) the DEGs between nectar and pollen foragers in our study with those identified by Whitfield et al. [52], and 2) the DEGs between pheromone treatments in our study with those identified by Alaux et al. [36] . We found significant overlaps between DEGs identified in our results and those of Whitfield et al. (*P* < 0.001) and of Alaux et al. (P < 0.001). The significant overlap between our study and the two microarray studies, which validate the expression patterns related to foraging specialization and brood pheromone exposure, suggests that foraging-related gene expression shows a degree of consistency across time scales (see [59]), and supports the idea that pheromones regulate the transcriptional pathways underlying foraging specialization.

Our data supported the hypothesis that exposure to larval pheromones alters expression of foraging related genes depending on foraging task specialization, but contrary to our prediction, the pheromones had more pronounced effects on gene expression in nectar foragers than pollen foragers (prediction 4). Larval pheromones have been shown to elicit specific responses in pollen foragers. For example, exposure to brood pheromone (BP) increased colony-level pollen foraging 2.5 fold [42], the ratio of pollen to non-pollen foragers [44], and the individual effort of pollen foragers [44]. However, prior to this study, there were no documented impacts of exposure to brood pheromones on nectar foraging. There was a common set of DEGs that were associated with both pheromone treatment and foraging specialization (Table 3), which was driven primarily by DEGs in nectar foragers but not pollen foragers (Table 4). Hierarchical clustering analysis showed that, for the most part, samples were clustered into pollen and nectar foraging “branches,” with the intriguing exception that nectar foragers exposed to EBO had expression profiles that were more like those of pollen foragers (Table 4). Similarly, PCA showed that nectar foragers exposed to EBO clustered more closely with pollen foragers than other nectar foragers (Fig. 3). The gene network analysis revealed that two modules were associated with both pheromone treatment and foraging, one of which was enriched for membrane components and energy metabolism (Table 8). These results suggest that one mechanism by which larval pheromones modulate colony-level pollen foraging behavior could be by downregulating metabolic pathways in the nectar forager brain, which is consistent with the role that energy metabolism plays in the ontogeny of foraging behavior [60]. Pankiw et al. [44] found that short exposure to BP increased pollen foraging, but did not observe task-switching of nectar foragers to pollen foraging, which the authors found puzzling. Our results indicate that one explanation may be that even after short exposures to larval pheromones, nectar foragers are primed to switch to pollen foraging even before they actually make the behavioral transition, which may be a way to buffer against ephemeral swings in the nutritional demands of developing larvae.

DEGs and WGCNA modules related to both pheromone treatment and foraging specialization were enriched for several metabolic pathways, including fatty-acid metabolism, but not epigenetic pathways, which suggests that metabolic processes and lipid signaling in integration centers of the honey bee brain may play a role in behavioral plasticity. The transition from nursing to foraging involves large-scale changes in metabolic pathways, including reductions in lipid stores and changes in insulin signaling [61]. These physiological changes during the transition from in-hive tasks to foraging are associated with changes in energy metabolism (including insulin signaling), gustatory response, and foraging preferences for nectar vs pollen [62, 63]. Therefore, the prominence of energy metabolism, lipid signaling pathways, and related metabolic pathways in our study’s brain transcriptome data supports the idea that these pathways in the brain play a role in insect behavior [64, 65]. Other studies have demonstrated the importance of brain metabolic processes on influencing individual variation in behavior, particularly aggression [65–67]. The enrichment of metabolic pathways in DEGs and the prominence of the FOXO signaling pathway in our gene co-expression networks further supports the role of insulin signaling pathways in mediating neuronal function and behavior in insects [64, 65]. For example, an insulin binding protein, *Queen brain-selective protein-1 (Qbp-1)*, was differentially expressed in response to pheromone treatment and is related to FOXO signaling. Module 3 was enriched for FOXO signaling and significantly correlated with EBO treatment, so its hub genes may serve as useful candidate genes for subsequent studies investigating the impact of insulin signaling on pheromone communication and foraging.

Although the results of this study are consistent with the interpretation that pheromone communication may possibly regulate foraging task specialization, the sample sizes on which this interpretation rests are relatively small. The consistency of the expression differences between our study and previous studies [36, 52], the patterns obtained in the PCA, and the results of the unsupervised clustering strategies (WGCNA & hierarchical clustering) all serve to indicate that our data may reveal biologically meaningful patterns despite the small sample size. However, future studies will be required to assess whether any confounding factors—such as individual variation among foragers, seasonal variation within a single colony, or variation among colonies—may influence the relationship between pheromone communication, foraging behavior, and regulation of gene expression. EBO has been shown to increase in non-pollen forager activity after pheromone exposure, despite within-day variation (i.e., between morning and afternoon trials) [46], which suggests that measurements of foraging activity are sensitive to temporal variation and sampling effects and that larval pheromones may produce increases in foraging activity that are robust to such temporal variation. The extent to which pheromone regulation of gene expression and behavior is dependent on such environmental factors remains to be evaluated.

The present study did not measure the aggregate colony-level foraging activity and focused instead on changes in gene expression within individuals. Previous studies demonstrated that larval pheromones increase colony-level pollen foraging behavior using an identical experimental paradigm for pheromone treatments [44–46, 51]. Larval pheromones have the greatest effect on pollen foraging for the first 3 hours following exposure [44], so the present study sampled foragers as they initiated foraging during this critical time window. Sampling foragers removes them from the foraging labor force and could potentially bias quantification of colony-level foraging activity, and thus we did not quantify colony-level foraging activity. However, the experimental design allowed us to sample foragers with distinct preferences because we collected foragers that had consistently visited pollen or nectar feeders as they attempted to collect food resources after pheromone treatment. While the gene expression differences in this study are correlated to pheromone exposure and to foraging preference for pollen or nectar, we did not mechanistically demonstrate that these differences in expression drive behavioral changes.

The results of our study lay the groundwork for several intriguing lines of inquiry for future studies. First, exposing foragers to a short pulse of BP, which stimulates immediate foraging, regulates a similar set of genes as exposing bees to BP for 5 days, which modulates the transition from in-hive tasks to foraging. This result suggests that one of the ways in which BP potentially regulates foraging behavior is by priming the receptivity of nurse bees to foraging-related or social stimuli, even before they have made the physiological transition to foraging tasks. This could conceivably involve genes implicated in both foraging and division of labor (e.g., *Malvolio,* a manganese transporter) [68], or neurochemical regulatory pathways involving octopamine, which has been shown to modulate responsiveness to both foraging-stimuli and to BP [69, 70]. Furthermore, our results suggest the hypothesis that social pheromones upregulate pollen foraging by decreasing the expression of nectar foraging genes in the brain, and this would also be productive line of inquiry for future studies. An alternative hypothesis is that social pheromones have context-dependent effects on gene expression that depend on the individual’s recent or past experiences rather than pollen foraging per se. Because our study focused on foragers with experience collecting either pollen or nectar, we could not distinguish whether changes in gene expression were due to previous experience with pollen foraging or innate preferences. Distinguishing between these two hypotheses may be an interesting future direction of work. Lastly, our data suggest that rapid changes in brain gene expression in nectar foragers may happen prior to task switching to buffer against ephemeral environmental conditions. Short-term exposure to larval pheromones may “prime” nectar foragers to switch preferences to pollen foraging, and this switch could occur under conditions of prolonged exposure to brood pheromone. Thus, our study provides a framework for hypothesis-driven experiments examining the impacts of pheromone exposure on task specialization and division of labor.

## Conclusions

The neural circuits and molecular pathways underlying behavioral plasticity and task specialization are complex, and our study demonstrates that foraging behavior may be regulated in part by common suites of genes across time scales, from long-term behavioral plasticity (nurse to forager) to individual variation in task specialization (pollen vs nectar). Our study further confirms that pheromone communication has a profound effect on gene expression within hours of exposure, and more importantly, that social signals (i.e. pheromones) may invoke foraging-related transcriptional pathways to upregulate pollen foraging at both long and short-time time scales. Moreover, there seems to be an interaction between individual variation in task specialization and responses to social signals (i.e. pheromones), and these social signals seem to invoke brain energy metabolism to elicit foraging behavior. Because the mechanisms underlying foraging behavior are deeply conserved in animals, a detailed mechanistic understanding of foraging in honey bees may provide insights into mechanisms involved in division of labor in insect societies, foraging preference in animals, and complex behavioral phenotypes in general.

## Methods

### Animals and experimental design

We created single-cohort colonies (using same-aged workers) from a common source colony with a naturally mated queen to avoid differences in behavior and gene expression due to variation in age and genetic background; thus, all bees used in the study were half-sisters. Because workers performing any given task in a natural colony can vary widely in age, we constructed single cohort colonies using workers that emerged as adults within a 48-h period, minimizing differences in age and experience among individuals. After 1 week, some of the bees in single-cohort colonies transition quickly to foraging (Robinson et al. 1989).

Three such colonies—each provided with an identical starting population (1000 bees), honey and pollen resources—were placed in a large outdoor enclosure (approximately 20′ × 50’ft) at the Texas A & M University Riverside Campus. Each colony was provided with two frames: one frame laden with pollen and honey stores, and one empty frame. In addition to frames full of honey and pollen inside the colony, feeders full of 50% (w/v) sucrose solution and fresh pollen (collected from pollen traps on unrelated honey bee colonies) were placed in front of each colony daily. All colonies received pollen from a common source each day. For 1-h per day during the three-day period before pheromone exposure, bees that foraged on each resource were marked with enamel paint on their thorax each time they visited a nectar feeder or pollen feeder (Testors, Rockford, IL). Bees visiting nectar feeders were marked with blue paint, and bees visiting pollen feeders were marked with yellow. Only bees with multiple paint marks (at least 2) of a single color were used in this study because multiple same-color marks demonstrated consistent preference for pollen or nectar, respectively. Each colony was also provided a strip of queen pheromone (PseudoQueen, Contech Industries, Victoria, BC, Canada) to prevent colonies from developing a “queenless” physiological state and to control for the variation in queen quality that would inherently occur when using live queens. Colonies did not receive any frames containing brood. Although broodless colonies are not the default state of a colony, it is nevertheless a natural occurrence when queens die for any number of reasons [55]. The absence of brood controlled for the natural variation in brood pheromones that may have occurred with the presence of real brood and minimized the amount of beekeeping interference necessary to maintain identical colony conditions.

After 2 weeks, colonies were exposed to field-relevant dosages (5000 larval equivalents) of EBO, BP, or a paraffin oil control. We used a BP blend characteristic of older larvae that has been shown to strongly upregulate pollen foraging [44], as done previously by Ma et al. [46, 71]. The BP blend consisted of 5% methyl palmitate,18% methyl oleate, 8.5% methyl stearate, 6% methyl linoleate, 10.5% methyl linolenate, 7.5% ethyl palmitate, 21% ethyl oleate, 11% ethyl stearate, 2% ethyl linoleate and 10% ethyl linolenate [72], and synthetic versions of EBO and all BP components were commercially available (Sigma Aldrich). Five thousand larval equivalents of pheromone [49, 72], or 0.5 mL of paraffin oil control, were placed in a glass petri dish and placed underneath the colony through a small trapdoor that allowed pheromone treatments to be placed and removed without otherwise disturbing the colonies. A fine wire mesh over the trapdoor prevented bees from directly accessing the petri dish or pheromone treatments. Hive entrances were blocked during the one-hour period during which the pheromone treatment was applied, and any foragers outside the colony during that time were removed from the experiment when they landed on the blocked entrance. When the entrances were opened, forager bees (previously marked as nectar or pollen foragers, as described above) were collected as they landed on a pollen or nectar feeder, but before they initiated feeding. Six pollen foragers and six nectar foragers were collected from each colony and placed immediately in dry ice for later brain dissection. Subsequently, we pooled pairs of bees to generate the RNA samples. Thus, in total, there were three pollen forager and three nectar forager samples for each of the three colonies representing the control, BP and EBO treatments (Fig. 1; total number of samples = 18). Sampled individuals were stored at − 80 °C until they were dissected.

### Brain dissection

Insect mushroom bodies are considered important integration centers of the brain because of their role in multimodal information processing and their association with learning and memory [73–75]. These factors make mushroom bodies an ideal candidate brain region to investigate temporal dynamics of communication and behavior. Therefore, mushroom bodies of the brain were dissected from sampled individuals by placing them on dry ice to prevent thawing and degradation of transcripts, as in [33, 36]. However, in our study, the brains were not freeze-dried to facilitate dissection of the mushroom bodies. For each sample, RNA from two brains were extracted using the RNeasy Mini Kit (Qiagen, Valencia, CA) following the manufacturer’s protocol, and pooled RNA quantity and quality were assayed using a Qubit Fluorometer (Invitrogen, Carlsbad, CA). cDNA library preparation and sequencing were performed by the Genomic Sequencing and Analysis Facility at the University of Texas at Austin using an Illumina HiSeq 4000 (Illumina, San Diego, CA) sequencer. All 18 samples were barcoded and split across four lanes to control for sequencing bias. A total of 18 RNA-seq single-end 50 bp libraries were generated, with three libraries for each treatment group from each colony (Fig. 1).

### Data analysis

Reads were trimmed using Trimmomatic [76] to remove adapter sequences, low-quality reads, and short reads (< 36 bp). Kallisto software was used to build a transcriptome index based on a recently updated honey bee reference genome annotation (Amel_HAv3.1), and subsequently to quantify the abundance of transcripts represented in each sample [77]. The R package tximport was then used to import transcript abundances and generate a gene-level count matrix that was scaled to both library size and transcript length [78]. The transcript-gene correspondence was derived from the genome annotation using the R package rtracklayer [79] (Additional file 3: Table S3). The R package DESeq2 [80] was used to collapse technical replicates (i.e. identical samples across multiple sequencing lanes) and perform differential gene expression analysis with pheromone treatment, forager type, and the interaction of pheromone treatment and forager type as fixed effects in a generalized linear model. Only genes with an abundance of at least one transcript per million in all samples were used in the analysis. Genes whose expression differed between groups were considered differentially expressed when they had a false discovery rate (FDR) of <0.05. Gene ontology analysis was performed using the Database for Annotation, Visualization and Integrated Discovery (DAVID v6.8) to better understand the biological relevance of differentially expressed genes (DEGs) [81].

The expression patterns of DEGs were further analyzed by performing unsupervised hierarchical clustering (Fig. 2; Additional file 4: Figure S1) and PCA (Fig. 3) on gene expression data normalized through variance stabilizing transformation. Hierarchical clustering was performed in R and visualized using the pheatmaps package [82]. Genes were clustered using the Ward method and samples were clustered based on manhattan distance. Principal component analysis (PCA) was performed to find the linear combinations of genes that explained the maximum amount of variation in the data, producing a series of orthogonal factors (i.e. principal components). PCA results were visualized in ggplot2 [83].

### Gene co-expression network analysis

To generate a global unsupervised overview of the gene expression data, we utilized weighted gene correlation network analysis [58] to identify groups of co-expressed genes and assess the relationship between these groups and experimental treatments. WGCNA constructs networks of genes based solely on the similarity of their expression patterns and organized them into groups of co-expressed genes, called modules (Additional file 5: Figure S2). Module assignment is unsupervised and independent of sample trait information (e.g., pheromone treatment, forager-type), and subsequently, these gene modules can be correlated with traits of interests. In this way, WGCNA can supplement other genomic and bioinformatic methods to provide a more detailed view of molecular processes associated with traits of interest.

Variance stabilized gene expression data were grouped into modules based on similarity of expression patterns. Because genes within each module showed very highly correlated patterns, the first principal component of the genes within a module was used to represent the entire module (module ‘eigengene’). Then, these module representatives were correlated with sample traits using a generalized linear model, with forager-type and pheromone as fixed effects (Fig. 4). Minimum module size was set to 30, and deep split was set to 2. Modules were built with a standardized connectivity score of − 2.5, and module definition was based on “hybrid” branch cutting. A signed gene co-expression network was constructed with a soft threshold of 10. Modules were merged based on a cut height of 0.1.

### Overlap of differentially expressed genes with previous studies

Hypergeometric tests were used to assess whether there was a significant overlap of differentially expressed genes when compared to other studies. Specifically, we tested overlap with genes regulated by long-term exposure to brood pheromone [36] and genes that varied between nurses and foragers [52]. These two studies utilized microarrays containing approximately 5500 genes identified in an earlier genome assembly version. For consistency, microarray probes were mapped to current official honey bee gene set, as done in Khamis et al. [84]. The degree of overlap between our data and data from these two studies were assessed using hypergeometric tests in the base stats package of R.

## Additional files

Additional file 1: **Table S1.** Transcriptome assembly quality metrics averaged across four technical replicates per sample, given in numbers of sequences per sample and as percentages of original sequencing reads per sample. (XLSX 13 kb)

Additional file 2: **Table S2.** Entrez gene IDs of differentially up-regulated and down regulated genes in the context of pheromone exposure, foraging task-specialization, and their interaction. (CSV 454 kb)

Additional file 3: **Table S3.** Dictionary of transcripts, Entrez Gene ID, BeeBase ID, and Accession numbers for all transcripts in the study. (CSV 1759 kb)

Additional file 4: **Figure S1.** Hierarchical clustering with multiscale bootstrap resampling confirms that bees exposed to identical pheromone exposure and forager-type produced distinctive transcriptional profiles in honey bee brains. For each cluster, two *p*-values are displayed on edges, expressed as percentages. The red number on the left represents the Approximately Unbiased (AU) method, and the green number on the right represents bootstrap probability (BP). Red rectangles indicate significant clusters with AU values greater than 95, indicating strongly supported clusters. Samples names denote pheromone exposure (i.e. Control (X), brood pheromone (BP), and E-beta-ocimene (EBO), forager type (Pollen (pol) vs nectar (N), or and sample number (1–3). This analysis used all 533 DEGs identified in this study. (PDF 5 kb)

Additional file 5: **Figure S2.** Clustering of variance stabilized gene expression data during co-expression network analysis. Modules were formed independently of sample information, and the colors under the cluster dendrogram indicate the assignment of co-expressed genes to modules. “Dynamic tree cut” colors indicate original module assignments before merging similar modules (cut height 0.1), while “Merged dynamic” colors represent final module assignments after merging similar modules. (PDF 214 kb)

Additional file 6: **Figure S3.** The third and fourth principal components (PCs) are displayed, which represent 19% of the total variation. Each point represents a single sample. Shape represents pheromone treatment. Color represents pollen or nectar forager-type. The percentage of variation in transcript expression patterns explained by each PC is shown in the y-axis. (PDF 5 kb)

Additional file 7: **Table S4.** Top 5 hub genes for each WGCNA module. (CSV 1 kb)

## Data Availability

The raw RNA-Seq data were deposited in the NCBI Sequence Read Archive under SRA study number SRP188881 and BioProject number PRJNA528102: http://www.ncbi.nlm.nih.gov/bioproject/528102. In addition, the code used to analyze the raw data is available at: 10.5281/zenodo.3250243.

## Abbreviations

BP: Brood Pheromone
DEG: Differentially expressed gene
EBO: E-beta-ocimene
FDR: False discovery rate
GO: Gene ontology
KEGG: Kyoto Encyclopedia of Genes and Genomes
PC: Principal component
PCA: Principal components analysis
RNA: Ribonucleic acid
RNA-Seq: RNA sequencing
WGCNA: Weighted gene co-expression network analysis
