# Supplementary figures and images for: Honey bee (Apis mellifera) larval pheromones may regulate gene expression related to foraging task specialization
Source: BMC Genomics. 2019 Jul 19;20:592. doi: 10.1186/s12864-019-5923-7 (PMC6642498; doi:10.1186/s12864-019-5923-7)

# Cluster dendrogram with AU/BP values (%)

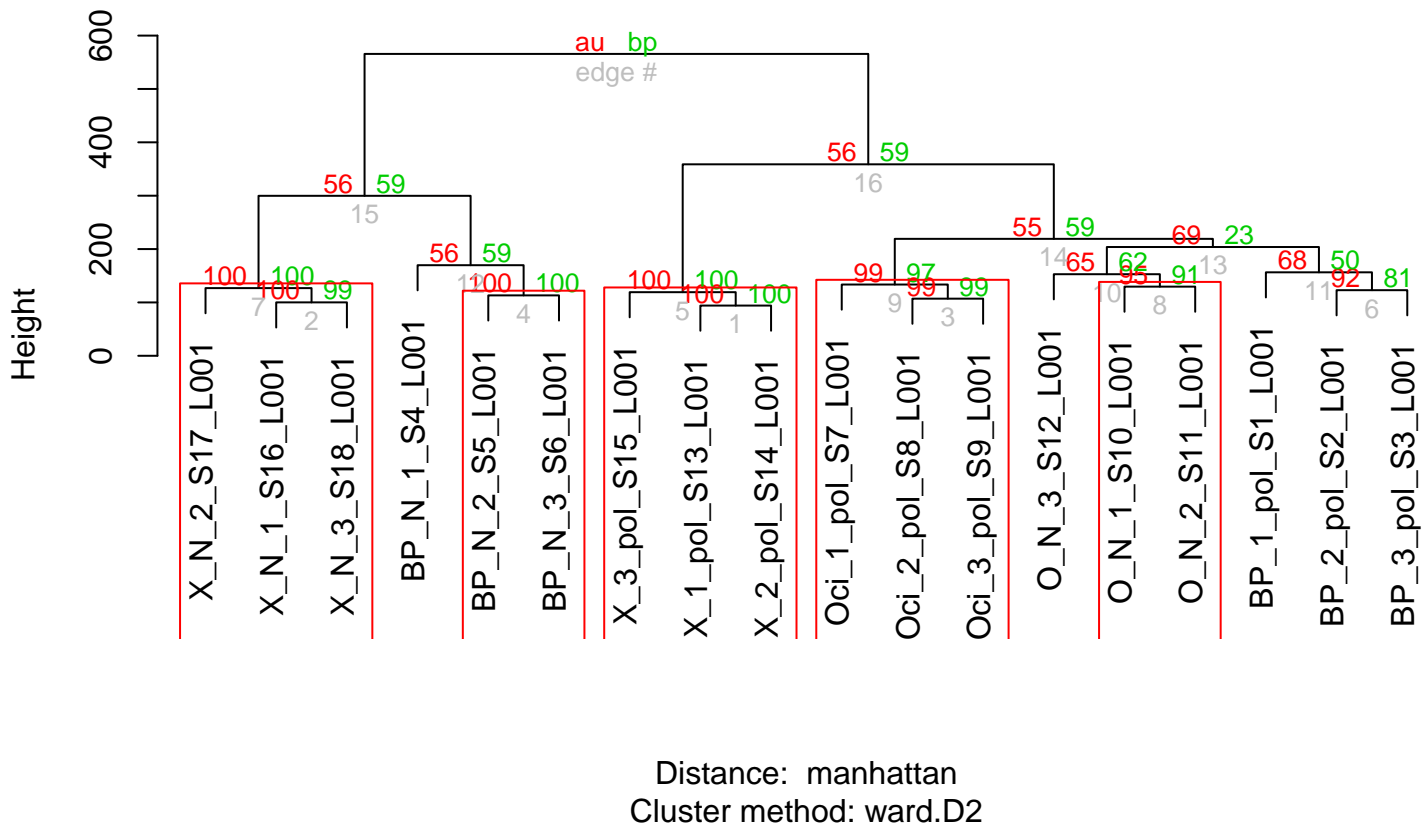

Supplement: Supplementary file 4 — Figure S1. Hierarchical clustering with multiscale bootstrap resampling confirms that bees exposed to identical pheromone exposure and forager-type produced distinctive transcriptional profiles in honey bee brains. For each cluster, two p-values are displayed on edges, expressed as percentages. The red number on the left represents the Approximately Unbiased (AU) method, and the green number on the right represents bootstrap probability (BP). Red rectangles indicate significant clusters with AU values greater than 95, indicating strongly supported clusters. Samples names denote pheromone exposure (i.e. Control (X), brood pheromone (BP), and E-beta-ocimene (EBO), forager type (Pollen (pol) vs nectar (N), or and sample number (1–3). This analysis used all 533 DEGs identified in this study. (PDF 5 kb) [file 12864_2019_5923_MOESM4_ESM.pdf]

# Cluster Dendrogram

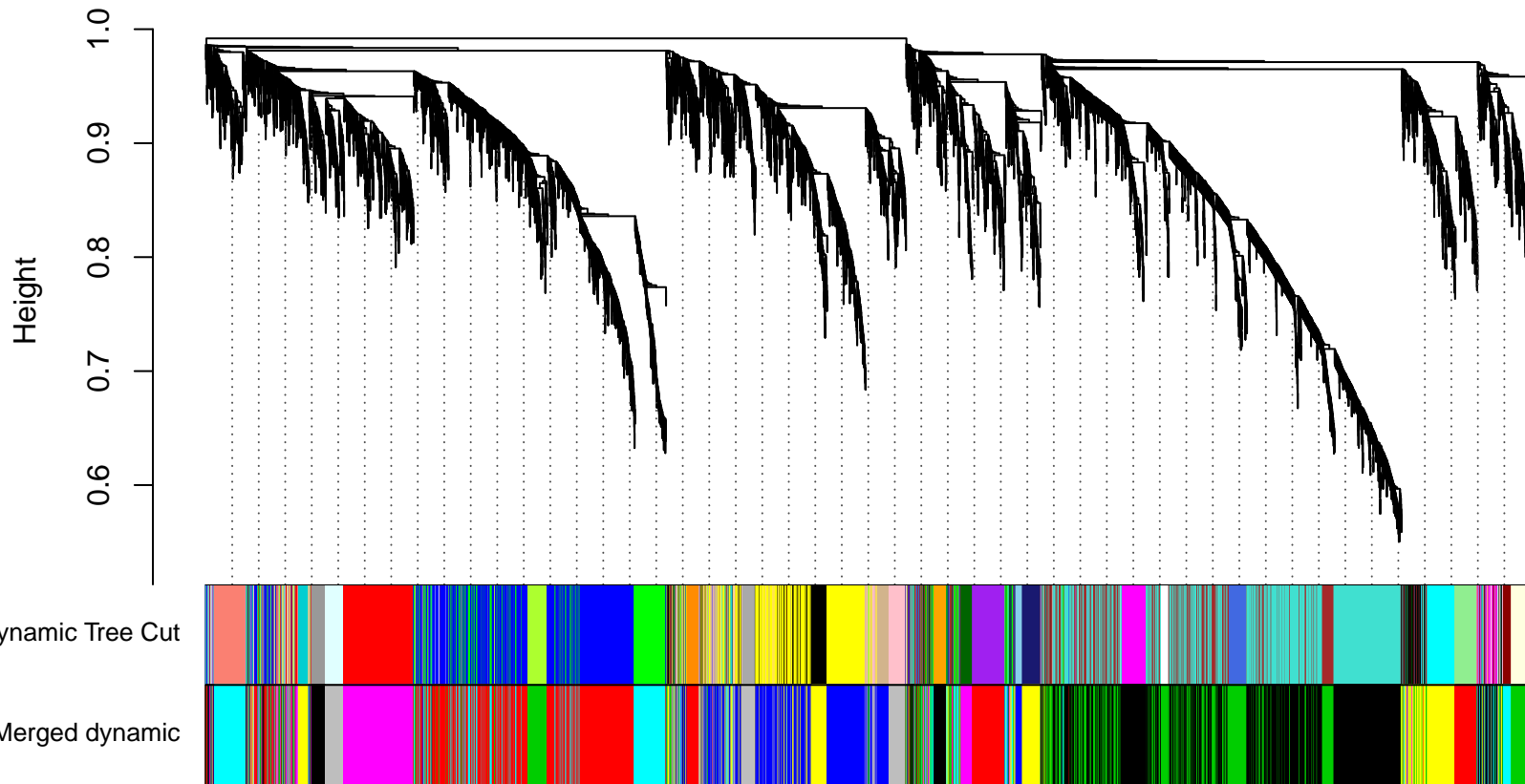

Supplement: Supplementary file 5 — Figure S2. Clustering of variance stabilized gene expression data during co-expression network analysis. Modules were formed independently of sample information, and the colors under the cluster dendrogram indicate the assignment of co-expressed genes to modules. “Dynamic tree cut” colors indicate original module assignments before merging similar modules (cut height 0.1), while “Merged dynamic” colors represent final module assignments after merging similar modules. (PDF 214 kb) [file 12864_2019_5923_MOESM5_ESM.pdf]

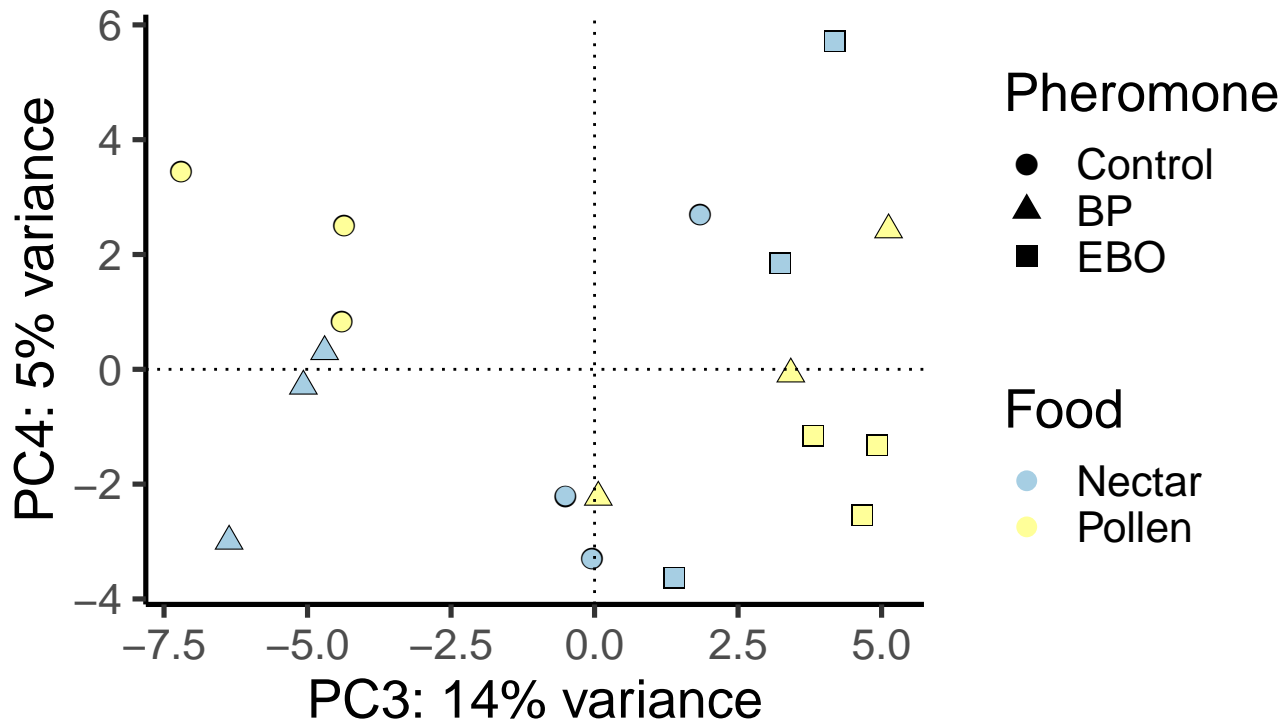

Supplement: Supplementary file 6 — Figure S3. The third and fourth principal components (PCs) are displayed, which represent 19% of the total variation. Each point represents a single sample. Shape represents pheromone treatment. Color represents pollen or nectar forager-type. The percentage of variation in transcript expression patterns explained by each PC is shown in the y-axis. (PDF 5 kb) [file 12864_2019_5923_MOESM6_ESM.pdf]
